# The Natural Products Atlas 2.0: a database of microbially-derived natural products

**DOI:** 10.1093/nar/gkab941

**Published:** 2021-10-28

**Authors:** Jeffrey A van Santen, Ella F Poynton, Dasha Iskakova, Emily McMann, Tyler A Alsup, Trevor N Clark, Claire H Fergusson, David P Fewer, Alison H Hughes, Caitlin A McCadden, Jonathan Parra, Sylvia Soldatou, Jeffrey D Rudolf, Elisabeth M-L Janssen, Katherine R Duncan, Roger G Linington

**Affiliations:** Department of Chemistry, Simon Fraser University, Burnaby, British Columbia V5A 1S6, Canada; Department of Chemistry, Simon Fraser University, Burnaby, British Columbia V5A 1S6, Canada; Department of Chemistry, Simon Fraser University, Burnaby, British Columbia V5A 1S6, Canada; Department of Chemistry, Simon Fraser University, Burnaby, British Columbia V5A 1S6, Canada; Department of Chemistry, University of Florida, Gainesville, FL 32611, USA; Department of Chemistry, Simon Fraser University, Burnaby, British Columbia V5A 1S6, Canada; Department of Chemistry, Simon Fraser University, Burnaby, British Columbia V5A 1S6, Canada; Department of Microbiology, University of Helsinki, 00014 Helsinki, Finland; Strathclyde Institute of Pharmacy and Biomedical Science, University of Strathclyde, Glasgow G4 0RE, UK; Department of Chemistry, University of Florida, Gainesville, FL 32611, USA; Strathclyde Institute of Pharmacy and Biomedical Science, University of Strathclyde, Glasgow G4 0RE, UK; Marine Biodiscovery Centre, Department of Chemistry, University of Aberdeen, Aberdeen AB24 3UE, UK; Department of Chemistry, University of Florida, Gainesville, FL 32611, USA; Department of Environmental Chemistry, Swiss Federal Institute of Aquatic Science and Technology (Eawag), 8600 Duebendorf, Switzerland; Strathclyde Institute of Pharmacy and Biomedical Science, University of Strathclyde, Glasgow G4 0RE, UK; Department of Chemistry, Simon Fraser University, Burnaby, British Columbia V5A 1S6, Canada

## Abstract

Within the natural products field there is an increasing emphasis on the study of compounds from microbial sources. This has been fuelled by interest in the central role that microorganisms play in mediating both interspecies interactions and host-microbe relationships. To support the study of natural products chemistry produced by microorganisms we released the Natural Products Atlas, a database of known microbial natural products structures, in 2019. This paper reports the release of a new version of the database which includes a full RESTful application programming interface (API), a new website framework, and an expanded database that includes 8128 new compounds, bringing the total to 32 552. In addition to these structural and content changes we have added full taxonomic descriptions for all microbial taxa and have added chemical ontology terms from both NP Classifier and ClassyFire. We have also performed manual curation to review all entries with incomplete configurational assignments and have integrated data from external resources, including CyanoMetDB. Finally, we have improved the user experience by updating the Overview dashboard and creating a dashboard for taxonomic origin. The database can be accessed via the new interactive website at https://www.npatlas.org.

## INTRODUCTION

Despite growing efforts to catalogue the known global secondary metabolome (e.g. COCONUT (1), LOTUS (2)), inconsistent dereplication methodologies and high rates of rediscovery still plague natural products discovery programs. (3). The Natural Products Atlas aims to address these issues by collating a standardized database of all known microbial natural product structures, source organisms and citations. This resource provides new discovery tools for the natural products community, including a user-friendly open-access platform for compound dereplication, and a standardized dataset of microbial natural product structures for new tool development. Recent tools that have incorporated the NP Atlas database include SMART 2.0 (4), NP Classifier (5), MIBiG (6), METASPACE (7) and the Natural Products Magnetic Resonance Database (www.np-mrd.org).

The original publication in 2019 describing the Natural Products Atlas contained 24 594 compounds and included a web interface for manual exploration of the data (8). In this new release we have increased the size of the database to 32 552 compounds, created a new API infrastructure and website to permit automated database queries, incorporated biosynthetic and small molecule ontology terms, and added full taxonomic descriptions of all source organisms, permitting filtering of the database at any taxonomic rank. Finally, we have performed an extensive re-curation of existing data to update structures with partial or missing configurational assignments. Together these advancements significantly improve the coverage, accuracy, and utility of this open access resource.

## METHODS AND IMPLEMENTATION

### Creation of Application Programming Interface (API)

A RESTful API for the underlying Natural Products Atlas database was created to facilitate extension and development of our web services, and to provide developers with facile access to the most up-to-date data and a suite of tools for automated complex queries. We have migrated our relational datastore from MySQL to PostgreSQL. This allowed us to leverage the RDKit PostgreSQL database cartridge extension, which provided full utility for chemical structure-based queries. Administrative functionality for creating, updating, and deleting data are included in an internal (non-public) version of the API, which also keeps a detailed changelog for improved data provenance. Additional custom search endpoints have also been added to simplify data queries from the Natural Products Atlas website. The FastAPI framework for Python was used to build the API. We provide a detailed OpenAPI specification and interactive documentation at https://www.npatlas.org/api/v1/docs.

### Addition of full taxonomic hierarchy

Every entry in the Natural Products Atlas includes the chemical structure, the original isolation reference, and the source organism from the original isolation. In the initial release source organism information was limited to the genus and species, as defined in the original publication. In the new version of the database we have incorporated additional data from Mycobank (9) and The List of Prokaryotic names with Standing in Nomenclature (LPSN) (10) to include assignments at all taxonomic ranks. This required us to refactor the origin tables in the database to accommodate terms for all taxa present in the compound database. Currently, these include: domain (3), kingdom (2), phylum (27), class (65), order (171), family (427) and genus (1,178).

**Figure 1.**
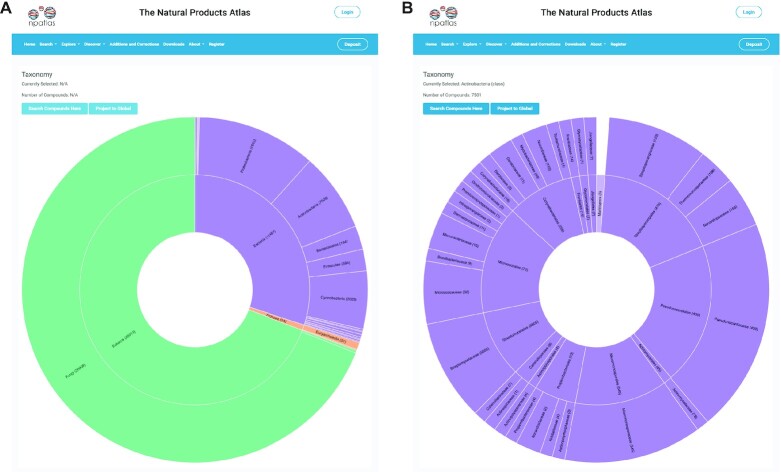
(**A**) Taxonomy dashboard screenshot. Each entry in the visualization may be clicked to view more detailed information about compound distributions within that taxonomic rank. (**B**) Visualization for all compounds of the bacterial class ‘Actinobacteria’.

To leverage this new information the search options in both the Basic and Advanced Search pages have been updated to accommodate filtering at any taxonomic rank. This can be combined with other search terms or structure or substructure searches to create custom search queries. In addition, we have created a new taxonomy dashboard that provides a visualization of compound diversity as a function of source organism taxonomy (Figure 1). Finally, the taxa in the Natural Products Atlas have been manually aligned against the NCBI taxonomy (11) and both NCBI taxonomy identifier (TaxId) numbers and links to Mycobank/LPSN entries are provided for each rank in the source organism section of the Compound page.

### Manual curation of additional database entries

The original release of the Natural Products Atlas included 24 594 compounds and covered a period up to early 2019. In this new release, we have expanded the database to 32 552 compounds, covering the period up to early 2021. This included targeted curation of 50 priority journals relevant to the field of natural products for 2019 and 2020, the insertion of new compounds submitted to the database through the deposition page on the website, and the integration of data from other external databases (see below). In addition, we continued our review of the historical literature to include additional compounds missed during the original curation effort. This included a retrospective review of existing data and the removal of 119 compounds from protists that are outside the scope of the Natural Products Atlas, as well as the removal of 51 compounds that were not of natural origin. Currently the alignment between the Natural Products Atlas and MIBiG 2.0 (a database of natural product biosynthetic gene clusters) is complete and up to date. It is our intention to maintain this alignment through ongoing bi-directional data curation for future MIBiG releases.

### Manual review of entries with incomplete configurational assignments

Retrospective evaluation of the database revealed that ∼10% of compounds were missing configurational information at one or more chiral centers. Because many of the original structures were derived from PubChem (12) or ChEMBL (13,14) rather than *de novo* transcription from the original papers into chemical drawing software we were concerned that configurational information may not have been captured in the original curation effort. To address this, we manually reviewed all articles describing compounds containing one or more undefined stereocenters (3154 compounds total) to verify the accuracy of the structures. This resulted in 487 updated structures, as well as the addition of 226 compounds not captured in the original curation effort.

### Incorporation of external databases

A valuable aspect of the Natural Products Atlas infrastructure are the options for user depositions and corrections. Although the number of reported corrections since the original release has been low, new depositions have been a steady source of information for the database. In addition, we have collaborated with external research teams to integrate data from various specialty collections. The largest of these efforts was the incorporation of the newly created CyanoMetDB database of cyanobacterial natural products (15). This included the alignment of structures and compound names between the two databases, review and correction of conflicts with source organisms and isolation references, and the addition of 800 CyanoMetDB compounds previously not present in the Natural Products Atlas. As with previous integration efforts, we have included CyanoMetDB ID numbers for all relevant compounds, permitting bidirectional navigation between the two resources.

Separately, Rudolf and co-workers recently performed a systematic review of terpenoid natural products from bacterial sources (16). We have collaborated with the authors of this review to integrate these data, including addition of 311 new compounds and corrections to structures and compound names for existing entries. Finally, we have also incorporated in-house compound collections from several academic research groups including the Müller laboratory at the Helmholtz Institute for Pharmaceutical Research Saarland (100 compounds) and the Clardy laboratory from Harvard Medical School (75 compounds).

### Addition of chemical ontology terms

Two different chemical classification systems have recently been developed for describing small molecule structures, NP Classifier (5) and ClassyFire (17). NP Classifier employs a biosynthetic ontology that is specific to natural products and is based on classifications that are widely accepted in the natural products community. By contrast, ClassyFire is a general classification system for small molecules (including organic molecules) that is based on the ChemOnt ontology. Both classification systems enable the subdivision of the database for targeted search applications, using terms of relevance to the natural products community. In this new release of the Natural Products Atlas, we have added classifications from both systems to every entry. These data have been added to the bottom of the Compound page and have been added as search terms in the Advanced Search page.

### Modifications to database structure

The original version of the database was built on a MySQL relational database that included capacity for a single compound name and source organism for each compound. Recognizing that molecules often have several synonyms in the literature, and that compounds are frequently reported from additional source organisms, we have refactored the database structure and the search engine to accommodate multiple entries for both fields. Currently, neither synonym terms nor additional source organism lists are complete. Work to identify and curate additional data in these two fields is an ongoing objective for the next database release.

### Creation of new website framework

The previous version of the database included a web interface built on the Content Management System (CMS) framework Joomla. Searches and interactive features were developed using a mixture of PHP and JavaScript (JS), and structure searches performed using a suite of custom tools within the Marvin chemical drawing plugin. Development of the new API provided an opportunity to simplify and modernize the website framework. We have removed the CMS layer and rebuilt the website from the ground up using the Django framework for Python with templated HTML pages and native JS for all interactive components and employing RESTful API queries for all search functions and database access. This has improved response times, and significantly simplified the framework for future development. For example, development of search pages and custom dashboards was particularly challenging within the CMS environment. This restriction has been removed with the refactor of the website, reducing the barrier to development for future data visualizations.

### Additional download options

The number of download options for the database has been increased in this new version. In addition to the original TSV download of the full database we now offer an Excel version of the same TSV format, an SDF download of all compounds, a structured JSON format for the full database, and graphML exports of both the Cluster and Node graphs displayed in the Explore section of the website. The SDF file is useful for importing into software that incorporates chemical structures, such as mass spectrometry and nuclear magnetic resonance data processing packages. The structured JSON is useful for developers who wish to recreate structured elements of the database without parsing the TSV file. The graphML files provide network representations of the chemical diversity of the database that were previously only available as interactive network visualizations in the web interface. Finally, previous versions of the database remain available to users via the Zenodo repository (https://doi.org/10.5281/zenodo.3530792).

## RESULTS AND DISCUSSION

### Data overview

**Figure 2.**
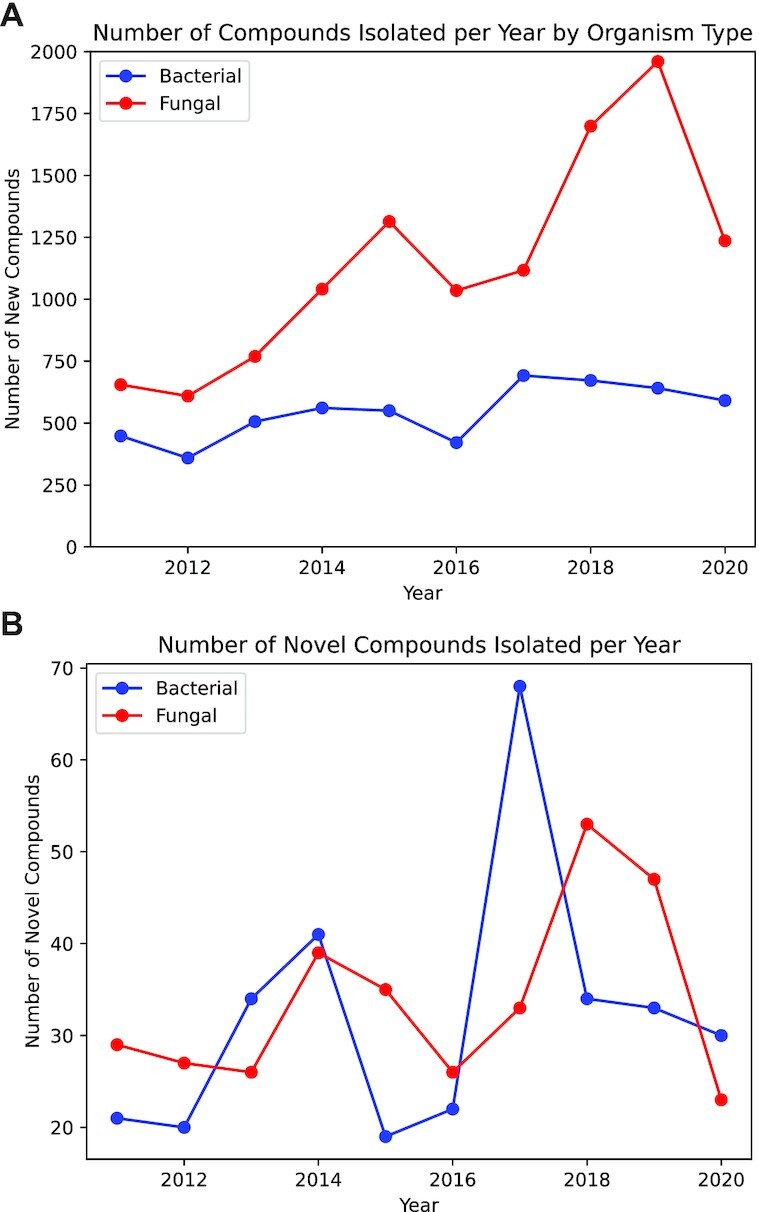
(**A**) Rates of compound discovery from bacterial (blue) and fungal (red) sources over the period 2011–2020. (**B**) Rates of ‘novel’ compound discovery from bacterial (blue) and fungal (red) sources over the period 2011–2020.

The new release increases the size of the database by 8128 compounds: 3176 of fungal origin and 4952 of bacterial origin. Over the past 10 years (2011–2020) the number of new compounds reported from bacterial sources has remained roughly constant at ∼540 per annum. However, the number of compounds reported annually from fungi has increased dramatically during this same period, from 655 in 2011 to 1236 in 2020 (Figure 2A). This has been accompanied by only a moderate increase in the number of publications on fungal natural products over the same period, from 212 in 2011 to 308 in 2020. This suggests that recent reports on fungal natural products are discovering more analogues per study than was typical in the previous decade.

Notably, the number of compounds with low structural similarity to known scaffolds has remained roughly constant at ∼60 compounds per year (Figure 2B). This is in line with our previous evaluation of compound novelty that demonstrated a steady rate of novel compound discovery in the presence of increasing rates of compound isolation. In this context, novel compounds are defined as those compounds which have maximum similarity scores <0.5 (Dice similarity, Morgan fingerprinting with radius 2) when compared to compounds from the same source type (bacterial or fungal) isolated in prior years. Somewhat surprisingly there is little difference between the rates of novel compound discovery from bacterial and fungal sources, even though many more fungal compounds are reported per year.

### Advantages of incorporating full taxonomic hierarchy

**Figure 3.**
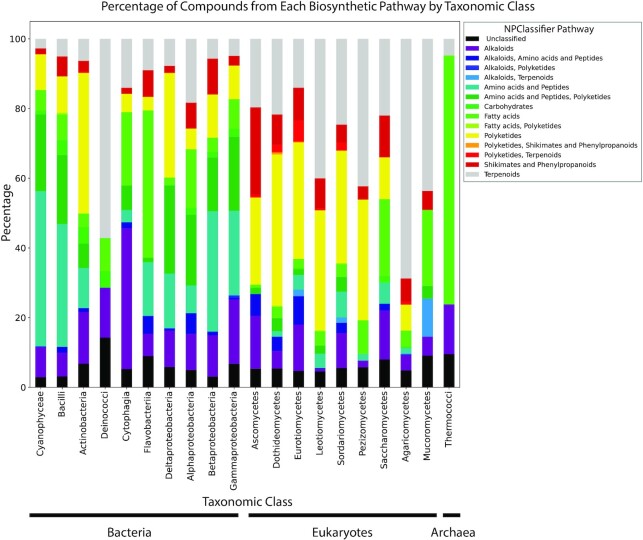
Percentage of compounds in each NP Classifier pathway separated by taxonomic class.

The addition of full taxonomic hierarchy and NP Classifier descriptions provides an opportunity to examine the distribution of compound classes across taxonomic space. Figure 3 presents the distribution of biosynthetic classes (NP Classifier pathway) by taxonomic class. Interestingly, there is strong consistency for the biosynthetic origins of compounds within each of the three domains (bacteria, archaea, and eukaryotes) but significant divergence in biosynthetic distributions between domains. For example, most bacterial classes are dominated by compounds of peptidic and hybrid polyketide synthase/non-ribosomal peptide synthetase (PKS-NRPS) origin, whereas most fungal classes include large numbers of polyketide and terpenoid natural products but few peptidic or PKS-NRPS structures. This is in line with other recent studies that have examined the biosynthetic distributions of compounds from microbial sources (18). Inclusion of these terms now permits users to filter search results by both taxonomic origin and biosynthetic class, enabling the creation of custom reference libraries for either category. This is valuable for research groups that study specific types of organisms (e.g. myxobacteria) and developers creating annotation tools for specific compound classes (e.g. DEREPLICATOR+) who require training sets for specific compound classes.

### Development of API and new website framework

The creation of a RESTful API further improves our commitment to FAIR (Findable, Accessible, Interoperable and Reusable) data principles, (19) providing improvements to two key facets: interoperability and reusability. Interoperability is dramatically improved by providing a persistent endpoint for other resources to automatically retrieve and query the latest version of the database. Power users have the added benefit of being able to construct complex queries and download large slices of data. From a reusability standpoint, the detailed changelog maintained by the API also provides much clearer data versioning and provenance.

The RESTful API was designed with four resources that are closely interrelated: compounds, references, taxa, and networks. These resources allow access to the data from all four perspectives, with compound entries linking all the data together. Supporting these four perspectives provides a clear path to users from various disciplines to be able to access or integrate data from the Natural Products Atlas into their own projects. For example, a taxonomy or BGC database is now able to automatically query and retrieve data from our API about which compounds were originally isolated from a given taxon at any level on the taxonomic tree. It also simplifies the development of novel dashboards and visualizations, such as our new taxonomy Discover dashboard (Figure 1).

### Legacy data review

Retrospective review of database entries can significantly improve database quality, particularly if existing data can be used to highlight ‘outliers’ for re-examination. However, re-curation of existing entries does not expand database coverage, and is therefore typically of low priority for academic database teams. Regular use of the Natural Products Atlas revealed that a small but significant number of compounds (∼10%) were missing configurational information at one or more chiral centers. This could either be due to incomplete configurational description at the time of discovery (more common for older papers), or inclusion of an incomplete structural representation from one of the public compound repositories (PubChem, ChEMBL etc.). Re-evaluation of these entries identified 487 natural products for which additional configurational information was available. More importantly, this re-evaluation validates the existing structural information, providing a strong foundation upon which to build future database development efforts and improving user confidence in the database contents.

A related issue is that natural product structures are occasionally corrected due to re-isolation and re-evaluation (20), computational reassessment of the original NMR data (21–23), or total synthesis (24,25). The Natural Products Atlas includes fields and search terms for reassignment data; however, the reassignment dataset remains incomplete. This is a complex task because structural reassignments are rarely the central objective of research studies. In consequence, these results are often not mentioned in article titles, making it time consuming to scan the literature for reassignment data. Development of tools to automatically identify articles reporting structure reassignments is ongoing in our group and forms one of the aims for the next cycle of database development.

An ongoing challenge with database development is the limited availability of machine-readable structure representations for new compounds. This significantly increases curation time due to the need to manually enter new structures and increases the number of structural errors. Recently, the Journal of Chemoinformatics has adopted a chemical structure data template (26) and has begun to encourage authors to deposit structure data as part of article submission (27). As noted by the proponents, this policy will greatly improve the FAIR properties of data from these articles. We hope that other journals will adopt this forward-thinking policy to improve access to chemical data and decrease the time required to incorporate new articles in to subject databases.

## CONCLUSION

The Natural Products Atlas has been structurally refactored to incorporate a new RESTful API and a new framework for the associated web interface. Within the database itself we have expanded the coverage of taxonomic information to include taxa at all levels and have added 8128 new compounds. These efforts extend current coverage to early 2021, while also backfilling legacy compounds that were omitted in the first iteration of the database and confirming or correcting the structures of all molecules missing configurational information at one or more chiral centers. Finally, we have added compounds from several custom databases, and have included ontological terms from two small molecule classification systems. Together, these improvements have expanded the range and scope of queries that can be performed using the web interface and provide new automated database access for developers of related external resources.

## DATA AVAILABILITY

The Natural Products Atlas is available at https://www.npatlas.org. The database is provided under a Creative Commons Attribution 4.0 International License (CC BY 4.0). Documentation for the API is available at https://www.npatlas.org/api/v1/docs.

## References

[1] Sorokina M. , Merseburger P., Rajan K., Yirik M.A., Steinbeck C. COCONUT online: Collection of Open Natural Products database. J. Cheminform. 2021; 13:1–13. 33423696 10.1186/s13321-020-00478-9 PMC7798278

[2] Rutz A. , Sorokina M., Galgonek J., Mietchen D., Willighagen E., Gaudry A., Graham J.G., Stephan R., Page R., Vondrášek J. et al. The LOTUS initiative for open natural products research: knowledge management through Wikidata. 2021; bioRxiv doi:28 May 2021, preprint: not peer reviewed 10.1101/2021.02.28.433265. PMC9135406 35616633

[3] Zdouc M.M. , Iorio M., Vind K., Simone M., Serina S., Brunati C., Monciardini P., Tocchetti A., Zarazúa G.S., Crüsemann M. et al. Effective approaches to discover new microbial metabolites in a large strain library. J. Ind. Microbiol. Biotechnol. 2021; 48:1–8. 10.1093/jimb/kuab017 PMC9113118 33599744

[4] Reher R. , Kim H.W., Zhang C., Mao H.H., Wang M., Nothias L.F., Caraballo-Rodriguez A.M., Glukhov E., Teke B., Leao T. et al. A convolutional neural network-based approach for the rapid annotation of molecularly diverse natural products. J. Am. Chem. Soc. 2020; 142:4114–4120. 32045230 10.1021/jacs.9b13786 PMC7210566

[5] Kim H.W. , Wang M., Leber C.A., Nothias L., Reher R., Kang K.B., van der Hooft J.J.J., Dorrestein P.C., Gerwick W.H., Cottrell G.W. et al. NPClassifier: deep neural structural classification tool for natural products. J. Nat. Prod. 2021; 10.26434/chemrxiv.12885494.v1. PMC8631337 34662515

[6] Kautsar S.A. , Blin K., Shaw S., Navarro-Muñoz J.C., Terlouw B.R., Van Der Hooft J.J.J., Van Santen J.A., Tracanna V., Suarez Duran H.G., Pascal Andreu V. et al. MIBiG 2.0: A repository for biosynthetic gene clusters of known function. Nucleic. Acids. Res. 2020; 48:D454–D458. 31612915 10.1093/nar/gkz882 PMC7145714

[7] Nguyen D.D. , Saharuka V., Kovalev V., Stuart L., Del Prete M., Lubowiecka K., De Mot R., Venturi V., Alexandrov T. Facilitating imaging mass spectrometry of microbial specialized metabolites with METASPACE. Metabolites. 2021; 11:477. 34436418 10.3390/metabo11080477 PMC8401310

[8] van Santen J.A. , Jacob G., Singh A.L., Aniebok V., Balunas M.J., Bunsko D., Neto F.C., Castaño-Espriu L., Chang C., Clark T.N. et al. The Natural Products Atlas: an open access nnowledge base for microbial natural products discovery. ACS Cent. Sci. 2019; 5:1824–1833. 31807684 10.1021/acscentsci.9b00806 PMC6891855

[9] Robert V. , Vu D., Amor A.B.H., van de Wiele N., Brouwer C., Jabas B., Szoke S., Dridi A., Triki M., Daoud S. Ben et al. MycoBank gearing up for new horizons. IMA Fungus. 2013; 4:371–379. 24563843 10.5598/imafungus.2013.04.02.16 PMC3905949

[10] Parte A.C. , Carbasse J.S., Meier-Kolthoff J.P., Reimer L.C., Göker M. List of prokaryotic names with standing in nomenclature (LPSN) moves to the DSMZ. Int. J. Syst. Evol. Microbiol. 2020; 70:5607–5612. 32701423 10.1099/ijsem.0.004332 PMC7723251

[11] Schoch C.L. , Ciufo S., Domrachev M., Hotton C.L., Kannan S., Khovanskaya R., Leipe D., McVeigh R., O’Neill K., Robbertse B. et al. NCBI Taxonomy: a comprehensive update on curation, resources and tools. Database. 2020; 2020:1–21. 32761142 10.1093/database/baaa062 PMC7408187

[12] Kim S. , Chen J., Cheng T., Gindulyte A., He J., He S., Li Q., Shoemaker B.A., Thiessen P.A., Yu B. et al. PubChem in 2021: new data content and improved web interfaces. Nucleic Acids Res. 2021; 49:D1388–D1395. 33151290 10.1093/nar/gkaa971 PMC7778930

[13] Davies M. , Nowotka M., Papadatos G., Dedman N., Gaulton A., Atkinson F., Bellis L., Overington J.P. ChEMBL web services: streamlining access to drug discovery data and utilities. Nucleic Acids Res. 2015; 43:W612–W620. 25883136 10.1093/nar/gkv352 PMC4489243

[14] Mendez D. , Gaulton A., Bento A.P., Chambers J., De Veij M., Félix E., Magariños M.P., Mosquera J.F., Mutowo P., Nowotka M. et al. ChEMBL: towards direct deposition of bioassay data. Nucleic Acids Res. 2019; 47:D930–D940. 30398643 10.1093/nar/gky1075 PMC6323927

[15] Jones M.R. , Pinto E., Torres M.A., Dörr F., Mazur-Marzec H., Szubert K., Tartaglione L., Dell’Aversano C., Miles C.O., Beach D.G. et al. CyanoMetDB, a comprehensive public database of secondary metabolites from cyanobacteria. Water Res. 2021; 196:117017. 33765498 10.1016/j.watres.2021.117017

[16] Rudolf J.D. , Alsup T.A., Xu B., Li Z. Bacterial terpenome. Nat. Prod. Rep. 2021; 38:905–980. 33169126 10.1039/d0np00066c PMC8107197

[17] Djoumbou Feunang Y. , Eisner R., Knox C., Chepelev L., Hastings J., Owen G., Fahy E., Steinbeck C., Subramanian S., Bolton E. et al. ClassyFire: automated chemical classification with a comprehensive, computable taxonomy. J. Cheminform. 2016; 8:1–20. 27867422 10.1186/s13321-016-0174-y PMC5096306

[18] Robey M.T. , Caesar L.K., Drott M.T., Keller N.P., Kelleher N.L. An interpreted atlas of biosynthetic gene clusters from 1,000 fungal genomes. Proc. Natl. Acad. Sci. U.S.A. 2021; 118:e2020230118. 33941694 10.1073/pnas.2020230118 PMC8126772

[19] Wilkinson M.D. , Dumontier M., Aalbersberg Ij.J., Appleton G., Axton M., Baak A., Blomberg N., Boiten J.-W., da Silva Santos L.B., Bourne P.E. et al. The FAIR guiding principles for scientific data management and stewardship. Sci. Data. 2016; 3:160018. 26978244 10.1038/sdata.2016.18 PMC4792175

[20] Sala S. , Nealon G.L., Sobolev A.N., Fromont J., Gomez O., Flematti G.R. Structure reassignment of echinosulfone A and the echinosulfonic acids A-D supported by single-crystal X-ray diffraction and density functional theory analysis. J. Nat. Prod. 2020; 83:105–110. 31934769 10.1021/acs.jnatprod.9b00902

[21] Ndukwe I.E. , Wang X., Lam N.Y.S., Ermanis K., Alexander K.L., Bertin M.J., Martin G.E., Muir G., Paterson I., Britton R. et al. Synergism of anisotropic and computational NMR methods reveals the likely configuration of phormidolide A. Chem. Commun. 2020; 56:7565–7568. 10.1039/d0cc03055d PMC7436192 32520016

[22] Tsui K.Y. , Tombari R.J., Olson D.E., Tantillo D.J. Reconsidering the structure of serlyticin-A. J. Nat. Prod. 2019; 82:3464–3468. 31840986 10.1021/acs.jnatprod.9b00859 PMC7187649

[23] Marcarino M.O. , Cicetti S., Zanardi M.M., Sarotti A.M. A critical review on the use of DP4+ in the structural elucidation of natural products: the good, the bad and the ugly. A practical guide. Nat. Prod. Rep. 2021; https://doi.org/10.1039/d1np00030f. 10.1039/d1np00030f 34212963

[24] Chong C. , Zhang Q., Ke J., Zhang H., Yang X., Wang B., Ding W., Lu Z. Total synthesis of anti-cancer meroterpenoids dysideanone B and dysiherbol A and structural reassignment of dysiherbol A. Angew. Chem.- Int. Ed. 2021; 60:13807–13813. 10.1002/anie.202100541 33847042

[25] Vieira De Castro T. , Yahiaoui O., Peralta R.A., Fallon T., Lee V., George J.H. Biomimetic synthesis enables the structure revision of littordials E and F and drychampone B. Org. Lett. 2020; 22:8161–8166. 33021803 10.1021/acs.orglett.0c03156

[26] Schymanski E.L. , Bolton E.E. FAIR chemical structures in the Journal of Cheminformatics. J. Cheminform. 2021; 13:1–3. 34229711 10.1186/s13321-021-00520-4 PMC8262078

[27] Guha R. , Jeliazkova N., Willighagen E., Zdrazil B. Reply to ‘FAIR chemical structure in the Journal of Cheminformatics’. J. Cheminform. 2021; 13:1–2. 34229726 10.1186/s13321-021-00521-3 PMC8261925

